# Intensive care unit (ICU) diaries and the experiences of patients’ families: a grounded theory approach in a lower middle-income country (LMIC)

**DOI:** 10.1186/s41687-020-00229-2

**Published:** 2020-07-23

**Authors:** Swagata Tripathy, Swati Priyadarshini Acharya, Alok Kumar Sahoo, Jayanta Kumar Mitra, Kishen Goel, Suma Rabab Ahmad, Upendra Hansdah

**Affiliations:** 1 Anesthesia & Intensive Care, AIIMS, Bhubaneswar, Odisha India; 2 Clinical Psychiatry, AIIMS, Bhubaneswar, Odisha India; 3 AIIMS, Bhubaneswar, India; 4 Trauma and Emergency Medicine, AIIMS Bhubaneswar, Bhubaneswar, India

**Keywords:** Qualitative research, ICU diary, LMIC, Patients’ families, Grounded theory, Mental health outcomes, India

## Abstract

**Objective:**

An intensive care unit (ICU) diary is a relatively new concept in low middle-income countries (LMICs). Illiteracy and socio-cultural inhibitions may affect the use and utility of this intervention, which has proven beneficial to patients and their families in high income countries (HICs). We aimed to explore how families of ICU patients experienced ICU diaries in our set up by using the Grounded Theory (GT) approach. A relatively new research tool, this enables exploration of a phenomenon to build theories in areas hitherto uncharted.

**Method:**

A clinical psychologist did 29 in-depth interviews of relatives of 13 patients admitted in the ICU for > 24 h for whom an ICU diary was being maintained. We used a three-step coding process- open, axial, and selective coding, followed by the formulation of a theory embedded in the data.

**Results:**

We found that the younger relatives of ICU patients accepted the idea better (age 30, SD 6) Half (48%) had education between 5th to 10th standards. Emergent themes suggested that for the family members, reading and writing the diary brought novelty, acted as a communication enabler, spiritual truss, and improved knowledge leading to change in perspective about the health care system. It also became a bridge to community bonding after patient discharge. Starting with appreciating the novelty of ‘diary entries,’ which was a new and exciting concept, family members used the diaries to communicate with health care workers (to gain information and understanding about the disease and treatment) and the patient to express their love and to maintain a connection. The diary acted as a confessional for hopes, fears, guilt, and faith for many members. As a tool, it enabled them to understand medical personnel as human beings and to appreciate their efforts, effectively improving confidence in the system. Finally, upon returning home, the diary was a crowd puller for extended family and neighbors encouraging discussions and enhancing bonding and information sharing.

**Conclusions:**

Our findings indicate a good acceptance of ICU diaries by family members in our ICU. With less literate, admitted ‘shy ‘members, in a society where ‘diary writing’ is not culturally rampant, the appreciation for the novel concept was universal. We see a place for these interventions not only at the patient/ family level but also as a means to ‘correct’ the image of health care workers in our society by humanizing ourselves to the end-user- the patient and his family.

## Introduction

Admission to an Intensive Care Unit (ICU) is recognized as a traumatic experience, both for patients and their families. Resultant mental health sequelae may take the form of anxiety, depression, or posttraumatic stress symptoms (PTSS) [1–4], adversely affecting quality of life (QOL) .

ICU diaries provide a day-to-day record of a patient’s admission to the ICU to his/her discharge. Taking the form of a narrative, they tell the story of patients’ stay in the ICU- in the words of the doctor, nursing officers, other healthcare workers (HCWs), and family members. Different ICUs vary in the design, content, and implementation processes of their ICU diaries.

Recent meta-analyses show that ICU diaries can improve psychological outcomes among ICU patients and their relatives [5, 6]. Their use began in Denmark in the 1980s; presently many ICUs in Europe implement ICU diaries [7].

The use of ICU diaries is uncommon in low middle-income countries (LMICs), and little is known, therefore, about how our population would accept them. There may be many barriers to adapting healthcare interventions that have proven useful in high-income countries (HICs) to LMICs [8]. Some common hurdles which come to mind would be the relatives’ inability to read or write, a cultural hesitation in expressing emotions on paper, the knowledge that others may read their feelings, and the difficulty of seeing photographs of their sick relatives. The engagement of HCWs and relatives of patients to meet in a common platform (the pages of a journal) is an unexplored area in LMICs.

The grounded theory (GT) approach is a structured qualitative research methodology appropriate in situations where little pre-existing knowledge exists. It involves collecting data in an iterative process, repeatedly returning to the ground (data) to generate a theory based on it [9]. It has been used previously to study the effect of ICU diaries on families of ICU patients in HICs [10].

As very little is known or published about the family’s experience with ICU diaries in LMICs, we performed a qualitative hypothesis-generating study. As part of a larger project in which we implemented diaries for our ICU patients, we aimed to explore how families of ICU patients experienced reading and writing in ICU diaries in our set up.

## Methods

### Context

We conducted the study in a 600 bedded tertiary care public hospital in Eastern India. The ICU is a 25 bedded mixed medical-surgical ICU. It has 24-h coverage by certified intensivists and post-graduate trainees. The nurse to patient ratio is 1: 2–3. Family participation in inpatient care is allowed as a part of the policy. All the patients were admitted to the same ICU and treated by the same critical care team. The Institute Ethics Committee approved the study, and relatives of ICU patients gave written consent for participation and use of their data (including photographs in the diaries) in research related academic activities.

**Fig. 1:**
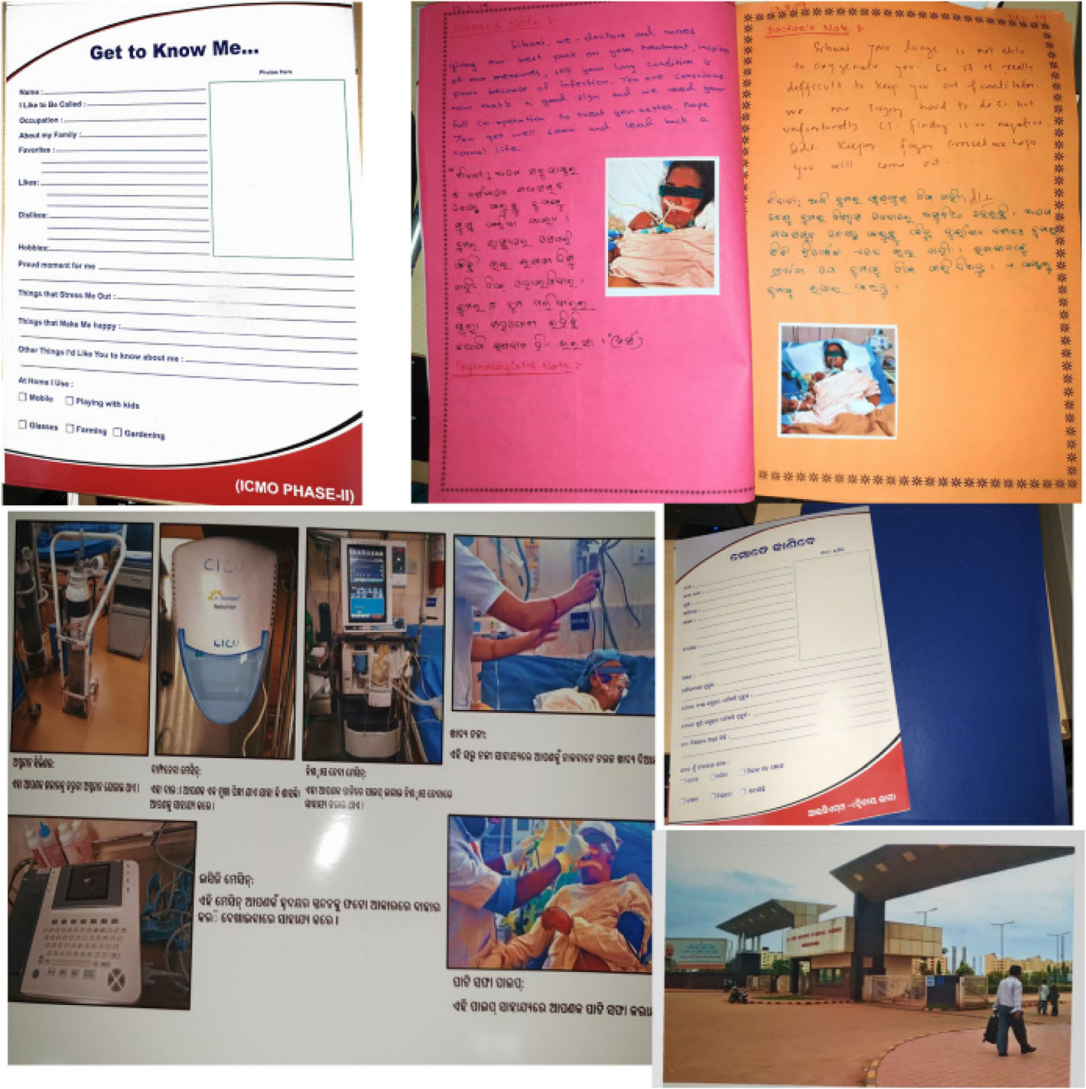
Representative Patient Diary

It was part of a larger project which aimed to assess the mental health outcomes of ICU patients and the effect of ICU diaries on the outcomes. We first designed and implemented a simple prototype. We met after the first five patients to finalize the design, layout, and implementation plan of the diary by taking feedback from stakeholders. We used colorful writing paper, multi-colored pens, and decorated the diary with pictorial cut-outs to make it attractive. A ‘Get to know me’ sheet with patients’ details such as a nickname, favorite food, and music was the standard first page. We included representative photographs (with explanations) of the hospital, the ICU, the standard equipment used, common procedures like nasogastric feeding, and suctioning in all diaries. We also finalized the implementation policies at this time. The diaries were started on the unit from April 2018. (An example of a typical diary, Fig. 1).

#### Implementation of ICU diaries

The research team had a series of lectures and meetings among doctors and nursing staff regarding the diaries. Emphasis was on addressing the patient directly, being expressive, and avoiding medical jargon. We placed a printed booklet with information and instructions about ICU diaries in the nurses’ station. The doctors and nurses involved in patient care and as many relatives as possible were reminded to make daily diary entries for each patient. The HCWs were requested to encourage family members to read and write in the diaries. The researchers explained to all users of the diary that the entries would be read by others and, therefore, to be aware of privacy issues while making entries.

There was a single diary for each patient. It stayed with the clinical psychologist (SA) who was a part of the research team. After the first entry by the doctor, which mentioned the admission status, everyone was free to create entries, in English or Odia (the local language) during their duty hours. Each entry in English was translated into Odia below it by the bilingual psychologist. Likewise, she also turned the Odia entries made by patient relatives into English. This would enable full use by all members of the family even after the patient was at home and allow the healthcare personnel to understand the family’s point of view. Many relatives requested support for writing in the diaries- their dictations would be directly transcribed into the diary by SA.

We maintained diaries for all ventilated patients who had prespecified risk factors. The diary was never left bedside as we were unaware of the impact of doing so. As a part of the study protocol, the diary was sent to the patient’s home by post at the end of 1 or 3 months after ICU discharge.

To collect data from the relatives of patients, we created an interview guide. We based the primary interview guide loosely on the one used by Oregas et al. [10]. Reflective and feeling-oriented open-ended questions followed informative questions.

#### Data collection

We conducted 29 detailed semi-structured interviews of the relatives of 13 patients admitted to the ICU between October 2018 to May 2019. The researchers involved in the study were fluent in 4 languages. Relatives able to read/ write or converse in any of these were included. The chief investigator (ST) and the clinical psychologist (SA), both trained in qualitative research methodology, could invite the relatives for in-depth semi- directive interviews. Participants were selected based on theoretical sampling: relatives who were more likely to use the diary were included. We collected the age, gender, education level, and relationship (to the patient) of each relative.

The interviews were conducted by SA, hired specifically for the study, and not a part of the ICU team. The meetings were held after patient discharge from the ICU- on the ward or at home to capture the full impact of the diary experience – the journey from ICU to home. The clinical psychologist interviewed all consenting relatives who visited their patients during the ICU stay. Each interview lasted 30 to 45 min. It was conducted in peaceful areas and was recorded (or manual notes were taken based on the interviewees’ choice) for transcription.

We collected data from 19 relatives in the first round of interviews. This data helped to create a provisional theory and modify the interview guide for the second round. The items were modified and altered with constant comparison as a part of the iterative procedure. In the second round, the questions on diary design were removed (as we reached data saturation). We included new items on the perceived impact of the diary on the knowledge of the healthcare system. We elaborated some questions from the first phase, which had elicited blunt responses such as yes/ no/all right to encourage more profound thoughts and answers. Ten additional family members were interviewed in the second round. The data collection method was similar to before. We list the questions used in the different phases of data collection in Table 1.

**Table 1:** The questions used in the two phases of the semi-structured interviews

**Family Questionnaire** 1. **Can you tell me how you used the dairy during your family member’s stay in the ICU?** 2. Can you tell me what you wrote and perhaps also why?* *Can you tell me what you wrote?* 3. How did it make you feel to write in the diary? * *Are you able to express your feelings completely? If yes, why? If no, why?* 4. Do you see a connection between how the diary was used and the course of your family member’s health condition? 5. Others will read what you wrote? How does that affect you? 6. Have you read what the staff members wrote during the ICU stay? How did that make you feel? *Do you feel any changes in your knowledge of hospital & ICU after writing the diary? *How does this knowledge help in a change in perception about the ICU Staffs, Doctors and Severe sickness changes? 7. Have you spoken about the diary to family members or friends? # *What is your opinion if the diary is kept alongside patients bed? *Will it be more useful to you or easier to read & write? *Do you think it should be sent home or not? *In-home, how is diary helping you, or the patient? 8. What do you think about the design of the diary? (Format, materials used, appearance) #	

Questions marked with a *were added or modified in phase 2: new question is in italics

Questions marked with a # were removed in phase 2

### Data analysis

**Fig. 2:**
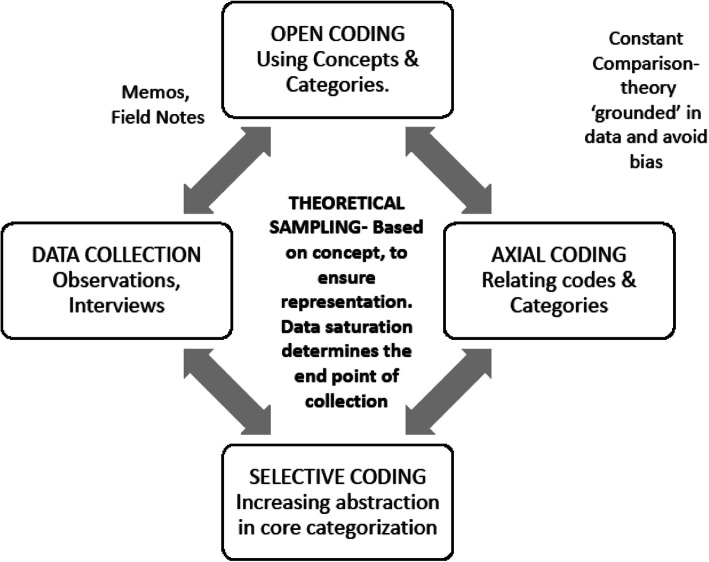
The Grounded Theory Approach. Stages of concurrent data collection and analysis

The GT approach was used [9, 11]. We used MAXQDA 2018 for data analysis [12]. Members of the team analyzing the data or involved in transcription and coding belonged to both ICU and non-ICU background, enabling an inside-outside technique. We read through the transcripts several times and began to create labels for chunks of data based on the meanings that emerged – open coding. Open coding involved labeling phenomena, discovering categories within them, and developing these categories. We relabelled subcategories as they emerged, comparing with new interviews and labeling in their own words (in vivo coding). Figure 2.

Then we identified connections among the various categories of open codes, recognizing relationships between them – axial coding. This enabled us to gain a more in-depth insight into abstract concepts and patterns in the data. Constant comparison with new data was made until no more changes could be introduced into the data structure.

Finally, we performed selective coding to integrate the categories and to identify the central category. The other categories and concepts were then related to the core to form a storyline. As per the dictates of the methodology, we moved back and forth between the collection of the data, analyzing it with deep immersion.

### Sample size

The sample size in GT is determined by the spectrum of data required to develop the theory (and not statistically determined as in quantitative research). It is determined by constant analysis of emerging data to identify new categories of participants or directions of query- and is impossible before data collection. It is acceptedly much less than in quantitative studies [11].

## Results

We interviewed twenty-nine family members of 13 patients (Table 2.) The patients had a median age of 46 (range 16) years, Acute Physiology and Chronic Health Evaluation score (APACHE II 14; range 2–18), length of ICU stay 8 days (range 4–16). Three patients had neurotoxic snake bite, 2 Guillain barre syndrome, two respiratory failure, four post-operative sepsis, one tubercular meningitis, and one was a polytrauma victim.

**Table 2:** Demographic details of the family members who were interviewed

Variable	N (%)
**Age (years, SD)**	30 (6)
**Male Gender n(%)**	19 (70.4)
**Relation with Patient n (%)**	
Spouse	3 (11)
Child	14 (48)
Sibling	12 (41)
**Occupation n (%)**	
Homemaker	8 (27)
Not Employed	2 (7)
Trade/ Shop keeper	10 (35)
Farmer	2 (7)
Manual worker	5 (17)
Office	2 (7)
**Education n (%)**	
Level 5 to 10	14 (48)
Beyond level 10	15 (52)

**Fig. 3:**
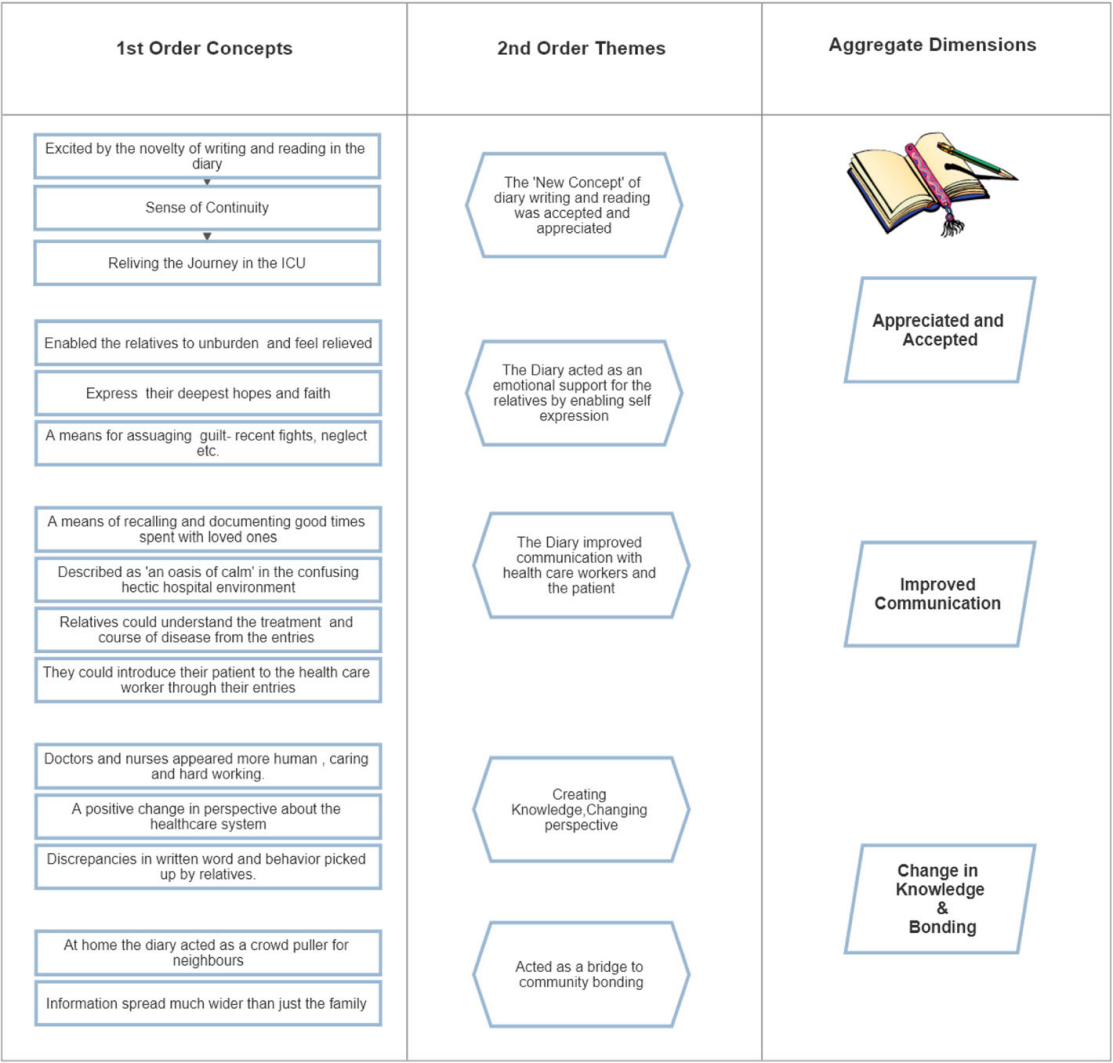
Data Structure

**Fig. 4:**
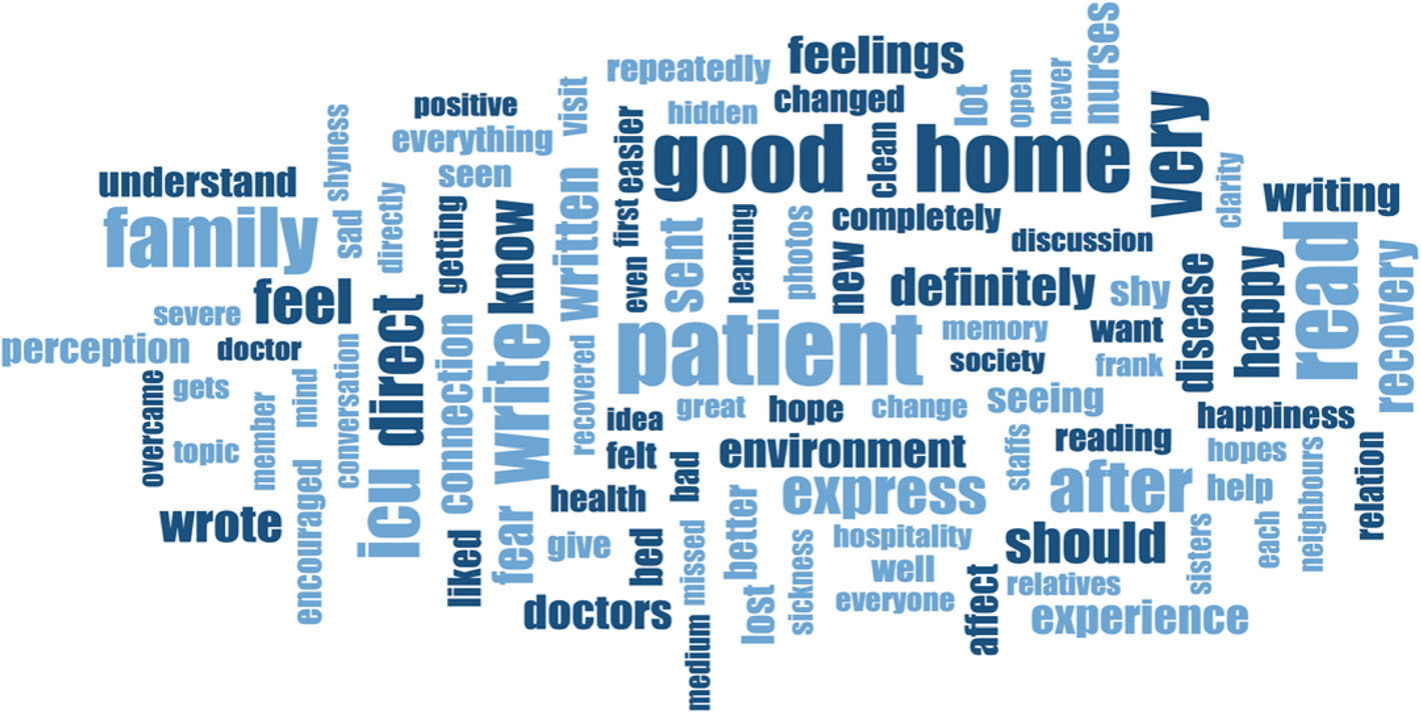
Relatives’ experience: reading and writing in the ICU diaries

The final data was made up of 15 first-order concepts, 5 second-order themes, and three aggregate dimensions (Fig. 3). Table 3 illustrates the number of people who contributed data to the different codes and categories. Figure 4 is a word cloud- a visual representation of the transcribed interviews.

**Table 3:** Number of participants who contributed data to creating codes and categories

Codes and Categories	No. of participants
**Diary Design**	**18**
**Sharing Information about the diary**	**25**
**Staff entries**	**12**
**Staff entry feelings**	**20**
**Effect of lack of confidentiality**	**26**
**New knowledge from using the diary**	**21**
**Connection with patient course- Information**	**22**
**Your feelings of writing- continuity/ reliving/ novelty**	**27**
**Why you wrote- Emotional Support/ Communication**	**18**
**Content of Diary- Documentation/ Introducing/ Expression**	**21**
**Use of Dairy- Neighbours/ Bonding/ Reliving**	**27**

### Appreciated and accepted

The diaries were liked universally by all the relatives (*n* = 29). The concept was new to them. Many found that it created a ‘sense of continuity’ even if they missed visits to the ICU and later when the patient was at home (*n* = 19). Relatives claimed to be able to express hitherto unsaid feelings towards their loved ones, feeling unburdened as a result (*n* = 18). Almost everyone mentioned that the diary was one place they used to express the deep faith and hopes of their patients’ recovery (*n* = 27).

A New Concept - All the participating relatives appreciated the diary as a new concept. Twenty-nine codes emerged from answers wherein the relatives expressed that they found this medium of expressing via writing one’s feelings a little daunting at first, not being used to the concept earlier. *“At first, I did not understand what madam wanted me to do. However, after reading the doctors’ and nurses’ entries, I could see what I had to do.”* (32-year-old wife) The unfolding of the events of every day provided a sense of continuity for the relatives, who compared the experience to television series/ magazine/ newspaper. The use of colored paper and pens and the decorations made the diaries intriguing to many relatives (especially the younger generation) *“ I loved the colored papers … I wish such a diary could be made for me!”* (15-year-old sister).

Facilitating Emotional Support- As the relatives got comfortable with using the diary as a source of information, sitting down with the diary to read the previous days’ entries, gave them a much-needed break. *“With this type of busy and hectic schedule, writing and reading the diary gave me consolation and calmness”* (42-year-old father) They could write in it to unburden their worries and even assuage their guilt. Many used it as a medium to recall good times spent with their loved ones and to express sincere hopes and faith. It got accepted by relatives. Table 4.

**Table 4:** Entries related to the ICU diary being Accepted and Appreciated

Themes	Concepts	Entries
**New concept**	**Appreciative the novelty of concept**	**1A BROTHER: I like the cover, protection design and photos.** **9A BROTHER: It’s a great idea and effort.** **15B SISTER: It is like a gift from the hospital.** **15C BROTHER: As it is a new thing, it feels good.** **15B SISTER: A new experience for me. I thank my sister (the ICU patient) that** **because of her I could experience something like this.**
	**Reliving (Photographs)**	**1C BROTHER: I liked my brother’s photo.** **Others: Reminded me how he came back from the ‘mouth of death’**
	**A feeling of continuity**	**3B SISTER: It had been printed like a magazine.** **Others: It is like a daily television serial, story unfolding.** **For me it is a newspaper telling me of my loved one** **A book about my brother!**
	**Exploring colours and creative writing material**	**9B SISTER: Colored papers are attractive. Creative writing material** **9B SISTER: Colored photos are attractive.** **9C SISTER: I have written in colored pen and paper like senior doctors and nurses.** **28B Daughter: I have written about the coloured paper and colored pen to my father.**
**Emotional Support**	**Unburdening Oneself**	**1C SISTER: As I could express myself so I felt light.** **3B DAUGHTER: Before and during writing I cried. After entry I felt unburdened.** **7A WIFE: I felt I could express my emotion and feelings directly in front of him.** **8A SON: I have written everything emotionally and this helped me feel light /unburdened.** **9B SISTER: I felt very happy and unburdened. But during writing I felt like crying.** **16B BROTHER: Felt relieved and unburdened by writing in it.**
	**Express Deep Hopes and faith**	**3B DAUGHTER: I wrote about father’s recovery and how I want father to get well soon.** **1B BROTHER: I just need him to be a good human and live a healthy life for his family.** **7A WIFE: I wrote that he should get better because I want him to improve with everyone’s efforts.** **7B BROTHER: I wrote encouraging words for my brother to get better.** **18A DAUGHTER: Hope of mother’s recovery.**
	**Assuage Guilt**	**18A SON: I had written all my feelings about my mother; I want to be a good son of her.** **31A DAUGHTER: Felt shy to write. If I had written everything, I would have asked for her forgiveness** **because I have many fights with mother, good or bad. When she was in ICU, I missed these times.**

### Improved communication

Enabling Holistic Communication- Reading the entries written by the healthcare workers brought out their humane sides for the relatives. *“I have read. I thought they were not treating a patient as an object but understanding him and trying to save his life”* (22 year-old-sister). Many wrote personal details about their patients because they thought it might help the health care workers form a bond with the patient and therefore treat him well. *“I do not mind if anyone reads what I write. If they (nurses and doctors) read my entries, they will understand him (relatives’ father) better”* (31-year-old son).

The diaries were also used as a means of communicating deep-seated emotions for the loved one- like a spouse expressing love. They also acted as a place to document information lost during ICU stay for the patient to catch-up after returning home*. “ I have to express my feeling towards my father in words. It is significant for me as it is a medium to know about my father’s health. I wrote all that happened during shifting him from home to hospital because he had missed it as he was not in sense.”* (38-year-old son). Table 5.

**Table 5:** Entries related to the ICU diary leading to Improved Communication

Themes	Concepts	Entries
With Healthcare workers	**Understanding the treatment and the course of disease** **Introducing & Associating the healthcare worker with the patient**	1C SISTER: They are giving time for the entries from their busy schedule. 3B SISTER: They had written in medical terminology about father’s care. 9B SISTER: I read all writing about treatment in my own language (odia). I felt good about it. 13A SON: They have written clearly about their efforts to save my brother. 17A Brother: I could understand all machines they use, how my brother is eating food through pipe in the nose … I felt at ease. 3A SON: I introduced my parent well on first page- if doctors know the wonderful person, they will treat him with full efforts. 15B SISTER: I get to realize how caring they are towards their patient from their entries. 16B BROTHER: They are writing truthfully and in a fair manner. 1A Brother: Nursing staffs writing was touching. 7A WIFE: I have read. I thought, they were not treating a patient as an object but understanding him and trying to save his life. 9A BROTHER: After reading I got to know that they are sensitive towards our feelings and our patients.
With Patients	**Documenting good times with loved ones**	3A SON: I have to express my feeling towards my father in words. It’s very important for me as it is a medium to know about my father’s health. I wrote all that happened during shifting him from home to hospital because he was not conscious and would not remember it later. 8A SON: Usually I am very straightforward and even rude to my father. But in the diary, I have written everything emotionally. 13A SON: I felt very happy. I have never expressed myself about anyone like this before. 15B SISTER: I wrote for her to get better. Because I am very concerned about her as she is my most favorite sister among all others. 17A BROTHER: I also wrote about the anxious situation at home so that she can understand when she reads it after recovery. 18B GRANDSON: I wrote good words for Maa. How I missed her when she is in ICU. 31A DAUGHTER: Felt shy to write. If I had written everything I would have asked for her forgiveness because I have many fights with mother, good or bad. When she was in ICU, I missed these times.

### Change in knowledge and bonding

Creating knowledge, changing perspectives- Once the relatives overcame their fear of expressing themselves on paper and embraced the ‘Novel Experience,’ the diaries became a place where they could document their decrease in fear of hospitals and appreciation of the health care system. *“A positive change has come. It has removed the fear of hospital, bad smell, and dirt because it was neat and clean.”.* The environment of increasing mistrust of doctors could be counteracted by the solidity provided by the written words of the hospital staff- *“I feel doctors are frank and stable. There is no hidden agenda. I felt they have humanity and compassion for us”.* (35-year-old wife) For some people, the diary was also a place they could see and compare discrepancies in the actual behavior and written word. We feel this was a starting point for quality improvement and constructive feedback, enabling community engagement in critical care- *“ … but if the nurses would behave as beautifully as they are writing, then the patient and the family will be happier.”*

### A bridge to community bonding

And finally, after the diary reached home by post, it was a source of comfort, joy, and awe to the recipients’ close family and neighbors alike. Neighbors saw the diary, and their knowledge about the critical care services and the medical system improved. Fear and ignorance were reduced. *“ I told everyone. They are eager and waiting to see how it looks after completion. We are showing it to everyone who comes to see my brother at home. We tell that it is a gift from the hospital to us.”* (29-year-old brother) Table 6. *“They were happy to see that the place where my father was kept was clean … bedsheets and blankets were provided. Doctors were always around!”* (31-year old daughter). *“ Our neighbors had heard that once a patient goes inside the ICU, he is good as dead … they were surprised when our brother returned after being bitten by the snake. They say if a snake bites anyone in their house, now they will go to the hospital*” *(26-year-old sister).*

**Table 6:** Entries related to the ICU diary leading to Change in Knowledge & Bonding

Themes	Concepts	Entries
Creating Knowledge, Changing Perspective	**Rebuilding faith in** **doctors and nurses** **Enables correlation between** **words and actions** **Changing perspective about** **Health Care System**	3B DAUGHTER: Yes, what they have told was written in the diary. Their words are very frank and supportive. 17A BROTHER: Doctors and nurses at AIIMS are handling severe sickness well. 28A SON: I feel doctors are frank and stable. There is no hidden agenda. I felt they have humanity and compassion for us. 31A DAUGHTER: I liked the doctors’ and nurses’ behaviour. 17A BROTHER: But if nurses could behave as beautifully as they are writing then the patient and the family will be happier. 13A SON: Whatever doctors are explaining to us was written clearly for my brother in the diary. 13B SISTER: A positive change has come. It has removed fear of hospital, bad smell and dirt because it was neat and clean. 18A SON: Impressed with hospitality of AIIMS Increased acceptability of severe sickness, staff and doctors. 28B DAUGHTER: After seeing repeatedly I feel AIIMS is a clean nice hospital. They give clean clothes and put my father on a bed! 28A SON: I overcame my fear of the hospital.
A bridge to community Bonding	**Exhibiting the Diary** **Spreading Knowledge about Health Care**	13 SON: I told everyone. They are eager and waiting to see how it looks after completion. We are showing it to everyone who comes to see my brother at home. We tell that it is a gift from the hospital to us. 15A HUSBAND: I had told everyone. Everyone was waiting eagerly for it to come to see it. 15B SISTER: I had discussed gladly about my and others staff members’ entries with my family and neighbors. 16A BROTHER: Everyone (in the extended family) was astonished when they heard about the diary at home. 16B BROTHER: Everyone was happy in our colony to see the diary. 3A SON: We can know the events which happened in the hospital. It will help us to be aware about the disease and hospital in the future. 3B SON: The family, relatives and neighbors can understand the treatment and hospital environment.

## Discussion

The concept of patient diaries is new in ICUs outside of the developed world. We investigated the experience of family members of ICU patients for whom ICU diaries were maintained. We found that the ICU diaries affected three broad areas. 1) The novelty of expressing oneself and ‘a personal touch‘of the HCWs was well accepted and appreciated as a new experience. 2) It improved family-staff and family-patient communication- information about daily health status was available regularly, to be read at one’s speed; the family could also speak about and introduce their patient, via the diary. The family members could communicate their hopes and emotions for their loved ones in the diaries 3) Family members reported improved knowledge and information about the critical care management of patients. Perspectives about HCWs and the system changed: they were humanized, seen as people who cared for their patients. These changes extended beyond the immediate family: due to a close-knit social fabric, the diaries were seen by whole neighborhoods once the patient returned from the hospital.

Our findings highlight some pertinent facts- diaries can be maintained in non- English-speaking populations where the doctors and staff mainly read and write in English (as in many south Asian ICUs). People who are not culturally used to writing in diaries can find the idea appealing and go on to use it for communicating, emotional support, and gaining knowledge, .

### Adaptation of diaries

The ‘Get to know me’ sheet used by previous researchers, allowed our HCWs to individualize their entries: this was appreciated by the family members [13]. Various recommendations by Chan-Dominy et al., such as the incorporation of a glossary of standard equipment and procedures in the local language and regular entries by doctors, have been used in other centers using ICU diaries [10, 14]. These worked well for us, as they improved staff-family communication.

What we did differently to adapt the diary design and implementation to our setting was

i. Routine translation of all the entries of the doctors and nurses into the local vernacular. This encouraged relatives to get involved. *“I read the entries in Odia. I liked the words.”* (32-year-old wife)
ii. We used colored papers, pens, and decorated the diary with pictures. There were photographs of the staff, relatives, and patients while in the ICU. This got good feedback as the family members likened the diaries to exciting colored magazine/ newspaper/ television series.
iii. Unlike previous studies, we had not kept the diaries on the patients’ bedside. All relatives agreed with this- most were concerned that the diaries might get misplaced. Two relatives expressed concern that if diaries are kept at the bedside in the ICU, it might scare the patient to see the photographs. After discharge, a few patients agreed that they would not have liked to see the diaries while in the ICU.

### Acceptance of the diaries

An observation of significance for us was that younger relatives (mean age of interviewees 30 + − 6 years) accepted and adapted to the idea faster. The older relatives, when approached, agreed to read the diary but hesitated to write or even dictate entries. All relatives who participated in our cohort were educated, but knowledge of the English language or higher education was not essential. This is different from a similar study by Garrouste-Orgeas et al., where the family members interviewed were aged 54.6 + − 13.0 years, interviewed only if French was their native language, and 75% of the family members had completed secondary level education [10]. Previous researchers have found that relatives treasure the diaries after reaching home: Storli and Lind report that relatives in their study considered the diary as a ‘caring gesture’ of the hospital staff, much like ours, who call it a ‘gift’ from the hospital [15]. Concerns about the negative aspects of ICU diaries have been raised by relatives previously, such as negative emotions like stress, guilt, failure, and exposure of intimate details [16]. Our group of family members expressed no such theme. The lack of confidentiality was also accepted well, with just 15% of relatives modifying their entries due to a privacy concern.

### Improved knowledge and bonding

The family members of an ICU patient seek information about the technological ICU world, the care their patient is receiving, and about the volatile and uncertain prognosis of their patient [17]. ICU diaries help in improving this information sharing and also humanizing HCWs [10]. A unique experience in our cohort, not mentioned in previous studies, was that once the diary reached the patient’s home, entire neighborhoods would gather around to see the diary and discuss the photographs and entries. Family members felt that this improved bonding among them and improved the awareness of people about the healthcare system. The diaries, therefore, also bridge the gap between the health care system and the end-user (patient, family, and community) by providing community engagement in the critical care journey. This may be an answer to improving communication and decreasing violence against doctors in the present Indian scenario [18].

Using an ICU diary has shown to decrease PTSS, anxiety, and depression among family members [19, 20]. Although our study did not assess the reduction in scores of mental health outcomes, many interviewees expressed feeling relieved, supported, and heard when they read and wrote in the diaries. *“It is now four weeks after my wife returned from the ICU. Even now, when we see the diary, we feel grateful to God that among other patients in the ICU who died, we survived and came back home. If those hard times are over, even these will pass …*.” (41-year-old husband).

The strengths of this study are using researchers with both ICU and non ICU background, having solitary and group meetings and returning to the data (grounding) repeatedly to allow a significant role and agency of the researcher in data construction and interpretation [21]. The limitations associated with the GT approach- the overabundance of data was handled well by using the MAX QDA software for data storage, collation, and analysis. We feel that using the GT approach was well suited to develop the preliminary theory, given the newness of ICU diaries in this part of the world. Doctors (consultants and trainees) and nurses participated in making the entries along with the family members. Theoretical sampling to data saturation enabled us to develop the theory in its entirety.

## Conclusion

Our study shows that the families of our patients received the idea of ICU diaries well. The diaries enhanced the ICU experience by creating bridges in communication, emotion, information, and hopes between the patient, relative, HCW, and the community. These easy, low-cost interventions may be encouraged as further data emerge. We feel that our study bodes well for future testing of this model in other LMICs.

## Data Availability

Data and materials can be made available from ST on reasonable request.

## Abbreviations

LMICs: Low middle-income countries
ICU: Intensive Care Unit
PTSS: Post traumatic stress symptoms
HICs: High Income Countries
GT: Grounded Theory
APACHE II: Acute Physiology and Chronic Health Evaluation II
